# Informed consent from patients participating in medical education: a survey from a university hospital in Jamaica

**DOI:** 10.1186/1756-0500-2-252

**Published:** 2009-12-15

**Authors:** Alan T Barnett, Shamir O Cawich, Ivor W Crandon, John F Lindo, Georgiana Gordon-Strachan, Diaqa Robinson, Deonne Ranglin

**Affiliations:** 1 Department of Surgery, Radiology, Anaesthesia and Intensive Care, Faculty of Medical Sciences, University of the West Indies, Mona, Kingston 7, Jamaica; 2 Department of Microbiology, Faculty of Medical Sciences, University of the West Indies, Mona, Kingston 7, Jamaica; 3 Research Centre, Dean's Office, Faculty of Medical Sciences, University of the West Indies, Mona, Kingston 7, Jamaica

## Abstract

**Background:**

Medical students at the University of the West Indies receive clinical training by passing through a series of hospital rotations at the University Hospital of the West Indies (UHWI). Many of these patients are unaware that medical students may be involved in their care. We performed this study to determine patient awareness and their willingness to participate in research and teaching activities.

**Findings:**

All consecutive patients admitted to the UHWI between May 1, 2006 and May 29, 2006 who required elective or emergency surgical procedures were prospectively identified These patients were interviewed using a standardised pre-tested questionnaire about their knowledge and willingness to have medical students participate in the delivery of their hospital care. Data was analyzed using SPSS Version 12.0.

There were 83 (39.5%) males and 127 (60.5%) females interviewed. The patients were unaware of the grade of the medical professional performing their interview/examination at admission in 157 (74.8%) cases or the grade of medical professional performing their operations in 101 (48.1%) cases.

Only 14 (6.7%) patients were specifically asked to allow medical students to be present during their clinical evaluation and care. When specifically asked, 1 patient declined. Had they been asked, 196 (93.3%) patients would have voluntarily allowed medical student involvement.

Only 90 (42.9%) patients were made aware that they were admitted to an academic centre with research interests. Only 6 (6.7%) patients declined. Had they been asked, 84 (93.3%) patients would be willing to participate in teaching or research projects.

**Conclusions:**

As medical educators, we are responsible to adhere to ethical and legal guidelines when we interact with patients. It is apparent that there is urgent need for policy development at the UWI to guide clinicians and students on their interactions with patients.

## Findings

The University of the West Indies (UWI) is the principal tertiary educational institution that caters to countries within the Commonwealth Caribbean [1]. As the only medical school in the Caribbean to be fully accredited by the Caribbean Accreditation Authority for Education in Medicine and Other Health Professions (CAAM-HP) in 2009 [2], the UWI plays a vital role to train health care professionals for the entire region.

At the UWI, medical students enter the undergraduate medical programme to read for the Bachelor of Medicine and Bachelor of Surgery (MBBS) degree over a period of five years [3]. Formal clinical training takes place during the latter three years, during which time medical students are passed through a series of hospital rotations and clinical attachments in the main medical and surgical disciplines [3]. During these rotations, medical students are exposed to patient care and are taught interviewing and examination skills [3].

At the Mona Campus of the UWI, the clinical rotations are carried out at the University Hospital of the West Indies (UHWI). This facility is a tertiary referral centre offering all medical and surgical sub-specialties [4]. Patients attending the UHWI benefit from exposure to highly trained academic physicians with a wide range of clinical and research experience. Many of these patients are, however, unaware that medical students may also be involved in their care to fulfil the requirements of the MBBS undergraduate degree programme. From an ethical viewpoint, these patients should be informed prior to their participation in medical education.

Much has been written on the principles of patient participation in medical education. One prevailing principle is that all patients have the right to decide whether they wish to participate in any medical educational activities through the process of informed consent [5-8]. Despite this, several authors have uncovered "gaps between principle and practice" that exist in teaching institutions regarding patient consent to participate in educational activities [9]. We performed this study to determine the awareness of patients admitted to the UHWI that medical students may be involved in their care and their willingness to participate in research and teaching activities.

## Methods

At the UHWI, patients presented to the Emergency Department when they required emergent medical attention. Patients who required non-urgent care presented to the ambulatory out patient clinics or directly to hospital wards for elective admission. Patients who were deemed to require in-patient care were admitted to hospital wards under the care of specific physician teams as demanded by their clinical presentation.

Approval was obtained from the local ethics committee at the UHWI to access patient records and interview patients for this study. We created a database of all patients admitted to the UHWI between May 1, 2006 and May 29, 2006. These patients were prospectively interviewed about their knowledge and willingness to have medical students participate in the delivery of their in hospital care.

We selected all consecutive adult patients who required an elective or emergency surgical procedure during their in-hospital care. These patients were selected for interview as a convenience sample because they should theoretically have significant patient-physician interaction during the process of securing informed consent for operation. Additionally, these patients would be subject to an invasive procedure that would likely involve medical students whether directly or indirectly.

Permission to participate in this study and to administer the study questionnaire was secured from the patients prior to their inclusion into the study. One of three investigators administered the questionnaires after being trained in administration techniques.

Patients were excluded from this study if they were: Under the age of 16 years; Deemed incompetent by virtue of their inability to hold a rational conversation for whatever reason (e.g. confused, tracheal intubation with sedation); Not able to sign their own consent form for whatever reason; or not willing to give informed consent to participate in the study.

A questionnaire was used as the instrument to collect information from patients in the database. The questionnaire sought information from the patients regarding their demographic information, socio-economic status, level of education, knowledge on their medical condition, knowledge of the qualification and training of the physicians and medical students involved in their care, and their attitudes toward being involved in medical education and/or research. Data from incomplete questionnaires were not included in the final analysis.

Data were analyzed using the Statistical Package for the Social Sciences (SPSS) version 12.0. Data were expressed as frequencies or means with standard deviations as appropriate.

## Results

During the study period, there were 263 consecutive patients admitted to hospital who required an operative procedure. After excluding 53 patients who met the exclusion criteria, there were 210 patients interviewed. This included 83 (39.5%) males and 127 (60.5%) females. Of this, 124 (59%) patients had already experienced at least one prior operation during a previous admission.

We found that 157 (74.8%) patients did not know the status of the medical professional who interviewed and/or examined them on admission to the hospital wards. They were also unable to differentiate between consultant physicians, post-graduate residents, interns and/or medical students who were involved in their care. Regarding their operative procedure, only 109 (51.9%) patients were aware of the identity and/or grade (medical student/post-graduate resident/consultant) of the medical professional who would perform their operation.

Of the total group, only 14 (6.7%) patients were specifically asked if they would allow medical students to be present during their clinical evaluation and the administration of their care. When specifically asked, 1 (7.1%) patient objected to the presence of medical students while they were examined and/or during any procedures that were required as a part of their medical care.

When the entire group surveyed was asked whether they would have allowed medical students to be present during their clinical evaluation and the administration of their care, 196 (93.3%) patients would have voluntarily allowed medical students to be present while they were evaluated. Of this group, 181 (92.4%) patients would allow medical students to participate unconditionally, 12 (6.1%) patients would allow medical students under the proviso that "they don't actively participate in their care" and 3 (1.5%) would allow them to be present "as long as there were not too many".

Regarding the potential for clinical information to be used in research projects, only 90 (4.3%) patients were made aware that they were admitted to an academic centre with research interests. When specifically asked, 6 (6.7%) patients voiced objections to their involvement in current or future research projects. There were 84 (93.3%) patients who were willing to participate in teaching or research projects, had they been asked to do so.

## Discussion

The UWI was founded in 1948 under the auspices of the University of London as a medical school with 33 medical students at the Mona Campus in Jamaica [1]. Since this time, the curriculum has expanded significantly and now includes all aspects of medical sciences that are taught at campuses in the Bahamas, Barbados, Jamaica and Trinidad [1].

In 2009, the UWI was the only medical school within the Caribbean to be fully accredited by the CAAM-HP [2]. This is a legally constituted body established in 2003 that bears the responsibility to determine standards and accredit medical school programmes on behalf of Caribbean Community (CARICOM) countries [2].

At the UWI, medical students study the basic medical sciences for two years, followed by clinical training for a further three years [3]. On completion of the fifth training year, a final qualifying examination is taken and the successful candidates are awarded a MBBS degree that entitles them to be provisionally registered with the medical councils of the English speaking Caribbean for internship [3]. Graduates then move on to independent practice or specialist training once they have worked in supervised internship posts for one year [3].

Medical students at the Mona Campus in Jamaica undergo formal clinical training at the UHWI. Here, they come into contact with patients in order to practice history taking and clinical examination skills [3]. This is usually done in a supervised fashion as a series of hospital rotations and clinical attachments to their tutors [3].

Patients being treated at the UHWI benefit from management by experienced and highly trained academic physicians. By participating in teaching and research, they also benefit by gaining access to experimental therapies [10-14], cost subsidization [15], increased availability of information [15-17] and closer attention when trainees participate in their care [11,15-17]. In turn, patients are expected to facilitate medical student training by allowing them to be present while they are being evaluated during the administration of care, to observe the performance of invasive procedures and to practice clinical skills.

Medical students gain invaluable clinical experience from the supervised participation in patient care [10]. In turn, the medical students are expected to maintain respect and observe patient confidentiality with all information gleaned from patients in the course of their care. They should clearly understand that they are not autonomous and are obliged to inform responsible clinicians regarding any information that they may elicit that is relevant to patient care.

We support the interaction of medical students and patients, as both parties benefit from the symbiotic relationship. However, medical students and their supervisors must be guided by ethical principles when interacting with patients. These include the principles of autonomy, beneficence, non-maleficence, and justice [18,19].

Unfortunately, we have demonstrated that the majority of the patients at the UHWI were not asked if medical students could participate in their care. Most patients did not even know the status of the medical staff conducting their interviews and/or examinations. This is not in keeping with the current ethical principles guiding physician-patient-medical student interaction.

Some health care workers have justified this by taking the stance that the Jamaican populace are generally aware that the UHWI is a teaching hospital. Therefore, by virtue of their attendance at the UHWI, patients have given 'implied' consent to the participation of medical students in their care.

Regardless of their knowledge of the background of the UHWI, patients do not waive their right to autonomy when they enter a teaching institution [20,21]. Apart from breaching ethical principles and the rights of the patient, we may also lose the trust of our patient, and instil in them feelings of fear and trepidation [22]. Additionally, patients often feel upset when they discover that they have been exposed to medical students without being informed [23].

On the other hand, patients are more accepting of medical students' involvement if their autonomy is preserved through the process of informed consent [23,24]. Even after patients are made aware of medical students' inexperience, they usually remain willing to partake in medical education, provided that they are treated respectfully [25] and with appropriate supervision [26]. This has been confirmed in our study, which found that most of our patients would not have objected to the participation of medical students in their care had they been asked.

Although there have been few reports of patients being opposed to medical student participation, particularly in obstetric and gynaecologic practice when it comes to male medical students [27,28], the overwhelming evidence suggests that the arguments for not informing patients seem to be based more on prejudice rather than evidence.

Several authorities have since outlined the importance of good ethico-legal practice governing medical student-patient interaction during training [9,29,30]. Doyal et al published the Patient Rights in Medical Education Policy in 2001 [9]. This document provides a detailed guide for clinical teachers to ensure proper medical student-patient interaction during training. The policies are readily transferable to the cultural and legal atmosphere at the UWI.

As medical educators, we are responsible to adhere to ethical and legal guidelines when we interact with patients. It is apparent that there is urgent need for policy development at the UWI to guide clinicians and students on their interactions with patients.

## Conclusions

Patients at the UWI are being inadequately informed and robbed of their legal and ethical right to autonomy regarding their participation in medical education. There is an urgent need for guidelines to be drafted, discussed and implemented in this setting. These steps would result in greater patient autonomy and facilitate the correct and optimal conduct of the clinical teachers.

## Competing interests

The authors declare that they have no competing interests.

## Authors' contributions

AB conceived the study, participated in its design and drafted the manuscript. SC participated in the study design, statistical analysis and drafting of the manuscript. IC participated in data acquisition, and helped to draft the manuscript. JL participated in data acquisition, study design and drafting of the manuscript. GGS participated in the study design, statistical analysis and drafting of the manuscript. DL1 and DL2 both participated in data acquisition and helped in drafting the manuscript. All authors have read and approved the final manuscript.
